# Audit of ‘As Required’ (PRN) Psychotropic Medication Prescriptions in an Adult Inpatient Psychiatric Ward

**DOI:** 10.1192/bjo.2026.11711

**Published:** 2026-06-30

**Authors:** Nihit Gupta, Jose Rodriguez

**Affiliations:** 1 Tees, Esk and Wear Valleys NHS Foundation Trust, York, United Kingdom; 2 Tees, Esk and Wear Valleys NHS Foundation Trust, Scarborough, United Kingdom

## Abstract

**Aims:**

PRN (pro re nata, or ‘as required’) psychotropic medications are commonly given by nurses based on patients’ needs to manage sudden psychiatric symptoms effectively. While these medications are essential in quickly relieving acute distress, inappropriate usage can cause issues such as excessive medication, unnecessary multiple drug prescriptions, and potential patient dependency. Therefore, strict compliance with prescribing guidelines is crucial.

This audit was conducted to check whether PRN psychotropic medication prescriptions on an adult psychiatric inpatient ward met the Trust’s guidelines (PHARM-0041-v4.2) and were prescribed safely.

**Methods:**

Retrospective audit of all PRN psychotropic prescriptions on Danby Ward, Cross Lane Hospital (TEWV NHS Trust) in the Electronic Prescribing and Medicines Administration (EPMA) system between 13 March and 5 April 2024 (registration 7482AMH23). Fifteen prescriptions comprising 32 PRN medicines were assessed against protocol-derived criteria (standard=100%): indication; dose/formulation/frequency; minimum dosing interval; maximum 24-hour dose; documented review date; first-/second-line guidance when more than one PRN option was prescribed for the same indication; and review/discontinuation if not administered for four weeks.

**Results:**

Indication and dosing parameters met the standard in all cases (32/32, 100%). No PRN medicine had a documented review date (0/32). Where more than one PRN option was prescribed for the same indication (18/32 medicines), first-/second-line guidance was absent in all applicable prescriptions (0/18; not applicable 14/32). Among medicines prescribed for four weeks or more without administration (8/32), none were reviewed or discontinued (0/8; not applicable 24/32).

**Conclusion:**

Non-compliance with PRN review standards is a medication-safety risk, with potential for delayed optimisation, avoidable polypharmacy/adverse interactions, and prolonged PRN use without a clear ongoing rationale. Improvements should focus on EPMA-enabled review prompts, routine pharmacy-led checks, targeted prescriber education, and clearer documentation to support consistent review and rationalisation.

Post-audit actions are largely completed Trust-wide: PRN oral and IM regimens now include an automatic 14-day review date; PRN guidance has been updated to clarify first-/second-line options and implemented across inpatient settings with updated EPMA content and training materials; EPMA has been upgraded to v3.3 to support Mental Health Act consent requirements; and EPMA e-learning plus a Quick Reference Handbook for medicines review/discontinuation have been produced. One action remains in progress: customisation of regimen dose screens, with change requests under discussion with Civica. The re-audit will assess compliance after full implementation.

